# MTHFR act as a potential cancer biomarker in immune checkpoints blockades, heterogeneity, tumor microenvironment and immune infiltration

**DOI:** 10.1007/s12672-023-00716-0

**Published:** 2023-06-24

**Authors:** Jianheng Peng, Zhongjun Wu

**Affiliations:** 1 Health Management Center, The First Affiliated Hospital of Chongqing Medical University, Chongqing, 400016 China; 2 Department of Hepatobiliary Surgery, The First Affiliated Hospital of Chongqing Medical University, Chongqing, 400016 China

**Keywords:** MTHFR, Cancer, Heterogeneity, Tumor microenvironment, Immune checkpoint blockades, Immune infiltration

## Abstract

**Purpose:**

To evaluate the role and landscape of 5-10-Methylenetetrahydrofolate reductase (MTHFR) to immune infiltration, tumor microenvironment, heterogeneity, immune checkpoints blockades, prognostic significance across cancer types.

**Methods:**

Data sets of genomic, transcriptomic and clinic features of MTHFR across > 60,000 patients and up to 44 cancer types were comprehensively analyzed using R software.

**Results:**

Expression of MTHFR gene is significantly lower in 17 tumors and correlated with overall survival (OS), disease-specific survival (DSS), progression-free interval (PFI) in specific tumors. Gene alterations of MTHFR are observed significant differences across tumor types. Expression of MTHFR is negatively correlated with the stemness index (mDNAsi, mRNAsi, DMPsi, ENHsi, EREG-mDNAsi and EREG-mRNAsi) in the most cancers. MTHFR showed significantly correlated with 67 types of immune cell infiltration scores in 44 cancer types by XCELL algorithm. Gene Ontology (GO) and Kyoto Encyclopedia of Genes and Genomes (KEGG) enrichment analysis are conducted to show the core tumor mechanism and biological process. Correlations between MTHFR and biomarkers of heterogeneity (MSI, TMB, MATH, HRD, LOH, Neoantigen, ploidy and purity) are also significant in specific tumors. MTHFR is significantly positively correlated with biomarkers of immune related genes (CD19, CD274, CD80, CD86) and mismatched repair genes (MLH1, PMS2, MSH2, MSH6, EPCAM, MLH3, PMS1, EXO1) in most cancer types. Receiver Operating Characteristics (ROC) analyses show MTHFR could act as a potential biomarker in anti-PD-1 (nivolumab to melanoma) and anti-CTLA4 (ipilimumab to melanoma) group of ontreatment, in anti-PD-1 (pembrolizumab to melanoma) group of pretreatment. Two immunohistochemistry antibodies HPA076180 and HPA077255 are verified in 20 types of tumor and could be used to detect the expression of MTHFR efficiently in clinic.

**Conclusions:**

MTHFR could predict the response of immune checkpoints blockades, heterogeneity, tumor microenvironment and immune infiltration.

## Introduction

The occurrence and development of tumor is very complex [1]. Genes [2], tumor microenvironment (TME) [1], immune infiltration [3], heterogeneity [4], et al., are all very important pathogenic factors. Moreover, precision therapy including immune checkpoint blockades (ICBs) and molecular targeted therapy of tumors have been emerging great benefit to patients but needing to make decisions based on the features of gene, molecular, protein and some relevant factors [5]. It is very urgent to find some specific markers to evaluate clinical prognosis and correlation between the potential mechanisms and therapy response.

5-10-Methylenetetrahydrofolate reductase (MTHFR) catalyzes the irreversible bioreaction of 5-10-MTHF to 5-methyl tetrahydrofolate, an active form of folate initiating methylation of homocysteine to methionine [6]. MTHFR gene including 11 exons and approximately 2.2 kilobases is on chromosome 1's short arm (1p36.3) [7]. MTHFR gene is involved in many biological processes, including Methionine biosynthetic process, the DNA synthesis, methylation and tumorigenesis [8]. Ranked top 10 most studied gene by Nature [9], MTHFR is prevalent in genetic test for clinic treatment. Two clinically identified MTHFR gene polymorphisms C677T [10] and A1298C [11], are the most researched. The C677T variant may occur with a range from 12 to 57% in European, Asian, American, and African populations [12, 13]. Both variants are associated with reduced enzyme activity [6]. Enzyme efficiency is reduced by up to 45% for the 677CT variant and by up to 70% for 677TT [14]. MTHFR deficiency causes homocysteine levels to rise, 5-methylTHF levels to drop, dysfunction of folate metabolism, DNA methylation and gene regulation [15]. MTHFR was researched to be associated with non-small cell lung cancer [16], breast cancer [17], esophageal cancer [18], gastric cancer [17], lymphocytic leukemia [19], hepatocellular carcinoma [20], thyroid cancer [21], among others. However, these discussions were continuing. But most researches have been concentrating on the relationship between the genetic mutation and different tumors, which has been showing positive, negative, or neutral associations across cancer types.

Until now, we still lack knowledge about the landscape of immune checkpoint blockades, heterogeneity, prognostic significance, stemness index, TME of MTHFR gene across cancer types. Thus We used the Cancer Genome Atlas (TCGA) project [22, 23], The genotype-tissue expression (GTEx) project [24] and the Gene Expression Omnibus (GEO) database [25] containing a large number of genomic datasets of tumors and normal tissues for Pan cancer analysis. We comprehensively characterized the gene alterations and transcriptome features of MTHFR across > 60,000 patients and up to 44 cancer types. Taken together, our study provided a valuable resource that will guide both mechanistic and therapeutic analyses of the role of MTHFR in cancer.

## Methods

### Expression analysis of MTHFR

We downloaded the unified and standardized pan cancer datasets TCGA TARGET GTEx (PANCAN, N = 19,131, G = 60499) from UCSC database, and integrate the expression data of ENSG00000177000 (MTHFR) gene in every sample. Each expression value was transformed by Log2 (x + 0.001). Finally, we rejected the cancer species less than 3 samples in a single cancer species. The RNA expression levels of 34 cancer and normal samples was calculated using the R software. An unpaired Wilcoxon rank sum and signed rank test were used to evaluate the significance of the difference. The interactive web UALCAN portal (http://ualcan.path.uab.edu/analysis-prot.html) for analyzing cancer Omics data was used to analyze protein expression of the Clinical proteomic tumor analysis consortium (CPTAC) dataset [26]. Additionally, We used R software (version 3.6.4) to calculate the difference in gene expression of each tumor in samples of different clinical stages. Unpaired Wilcoxon Rank Sum and Signed Rank Tests were used to analyze the significance of the difference between the two groups. Use kruskal.test to test the difference between multiple groups of samples. Each expression value was transformed by Log2 (x + 0.001).

### Survival prognosis significance of MTHFR

In addition, we also obtained a high-quality prognostic dataset from the TCGA prognostic study previously published on Cell [27]. Data on follow-up targets was retrieved from the UCSC cancer browser (https://xenabrowser.net/datapages/) as a supplement, and cancer species with less than 10 samples were rejected. Finally, we analyzed the data of overall survival (OS) of 44 tumors, disease-specific survival (DSS) of 38 tumors, Disease-free interval (DFI) of 32 tumors, progression-free interval (PFI) of 38 tumors. Relationship between MTHFR expression and prognosis of tumor patients was analyzed by using a Cox proportional hazards regression model applied in R software survival [28]. We calculated the optimal cut-off value of MTHFR expression by using R software package maxstat (Maximally selected rank statistics with several p-value approximations). A minimum of 25% and a maximum of 75% of grouped samples were set and the optimal cut-off value of each tumor sample was finally obtained. Besides, the prognostic differences between high and low expression groups were further analyzed by using the survivfit function of R software package. Logrank test was used to obtain the significance of prognosis.

### Gene alteration analysis of MTHFR

The Cancer Genomics web named cBioPortal (https://www.cbioportal.org/) is an important tool to explore and analyze cancer genomics data [29]. We entered “MTHFR” for queries of the gene alteration characteristics of MTHFR in the “Quick select” section of the “TCGA Pan Cancer Atlas Studies”. According to the “Cancer Types Summary” module, alteration frequency and type [structure variant data, mutation data and copy number alteration (CNA)] were displayed across all TCGA tumors.

Downloaded from GDC (https://portal.gdc.cancer.gov/), datasets of CNA (Fig. 3c) of gene level 4 of all TCGA samples were processed by MuTect2 [30] and gistic software [31] separately. The domain information of the protein was obtained from the R software package maftools. Each expression value transformed with log2 (x + 0.001). We eliminated cancer species with fewer than three samples.

### Machine learning identifies stemness features associated with MTHFR

We obtained six tumor stemness indices calculated by mRNA expression and methylation signature from previous studies [32], including transcriptomic RNA expression-based stemness index (mRNAsi), epigenetically regulated RNA expression-based index (EREG-mRNAsi) (103 genes) and epigenetic DNA methylation-based Stemness index (mDNAsi) that combines the 3 signatures listed below, epigenetically regulated DNA methylation-based index (EREG-mDNAsi), differentially methylated probes-based index (DMPsi), enhancer Elements/DNA methylation-based index (ENHsi). We identified 82 DNA methylation probes of the HM450 platform that mapped to enhancer elements and considered them to be a DNA methylation-based pluripotent stem cell enhancer signature, which was then used as input for the OCLR to evaluate stemness signatures for TCGA samples, named ENHsi [32]. A set of 62 pluripotent cell-specific and differentially methylated regions, which was then used as input for the OCLR to determine the stemness index for each TCGA tumor sample, named DMPsi [32].

### Immune infiltration analysis of MTHFR

We extracted the gene expression profile of each tumor and mapped the expression profile to GeneSymbol. Furthermore, deconvo XCELL [33] and deconvo_EPIC [34] of R software IOBR [35] was used to reassess the immune scores of each patient in each tumor based on MTHFR expression, including CD4+T cells, CD8+T cells, B cells, Immune Score, Stroma Score, Microenvironment Score, et al. We analyzed TCGA tumor data using the "Immune-Gene" module of the TIMER2 web server to examine the association between MTHFR expression and immune infiltrates. The XCELL, MCPCOUNTER and EPIC algorithms were applied for immune infiltration estimations. Purity-adjusted Spearman's rank correlation test was used to derive the P-values and partial correlation (cor) values. Heatmap and scatter plot were used to visualize the data.

### MTHFR related enrichment analysis

A search on the STRING website (https://string-db.org/) for “MTHFR” and “Homo sapiens” was conducted first. Subsequently, Following was the main parameters we set: the minimum required interaction score “Low confidence (0.150)”, meaning the network edges to be “evidence”, max number of interactors to choose “no more than 20 interactors” in 1st shell and active interaction sources “experiments”. Finally, A list of the experimentally determined MTHFR-binding proteins was compiled. Then, we downloaded the data of Gene Ontology (GO) and Kyoto Encyclopedia of Genes and Genomes (KEGG) Pathway in MTHFR-binding proteins network for enrichment analysis.

Based on the datasets of all TCGA tumors, we used the “Similar Gene Detection” module of GEPIA2 to identify the 100 most MTHFR-correlated targeting genes. Here we only select the 5 most related genes to show the relationship with MTHFR. Then, we used GEPIA2's “correlation analysis” module to determine the pairwise gene Pearson correlation of MTHFR and selected genes. Moreover, we used the “Gene_Corr” module of TIMER2 to supply the heatmap data of the 5 most related genes, which contains the partial correlation (cor) and P-value in the purity-adjusted Spearman's rank correlation test. We used Genecards tool to modify the symbol of the 5 genes.

For gene set functional enrichment analysis, the latest gene annotations of KEGG pathways were obtained using the R software package org.Hs.eg.db and KEGG rest API (https://www.kegg.jp/kegg/rest/keggapi.html). This set of genes was used as the background for mapping the genes. And the results of gene set enrichment analysis were obtained using R software package cluster profiler. P-value < 0.05 and FDR < 0.25 were considered statistically significant.

### Heterogeneity and MTHFR

Downloaded from GDC (https://portal.gdc.cancer.gov/), Dataset of single nucleotide variant (SNV).of gene level 4 of all TCGA samples was performed by MuTect2 software. R software maftools was used to analyze the Tumor mutational burden (TMB) and Mutant-allele tumor heterogeneity (MATH) of 37 tumors. Gene expression Data of Microsatellite instability (MSI) and homologous recombination deficiency (HRD) accessed from previous analysis also was integrated. Data of loss of heterozygosity (LOH), Neoantigen (NEO), ploidy and purity [36] also were recruited to demonstrate the correlation between MTHFR and the heterogeneity of tumors. Furthermore, TIMER tool was used to analyze the relationship between MTHFR and Nine mismatched repair (MMR) genes in tumors.

### Immune related genes (IRGs) and MTHFR

Expression data of MTHFR and 150 immune related genes, including chemokine (41 genes), receptor (18 genes), major histocompatibility complex (MHC) (21 genes), Immunoinhibitor (24 genes), Immunostimulator (46 genes), was extract from samples. Furthermore, we screened samples from primary blood derived cancer—peripheral blood and primary tumor, and calculated the Pearson correlation between MTHFR and marker genes of five immune pathways. For specifically, TIMER tool and Scatter plot were used to calculate the correlation between the IRGs such as CD19, CD274, CD80 and CD86 with MTHFR in tumors.

### Receiver operating characteristics (ROC) plotter validates response of ICBs

Differentially expressed genes can be used to classify biological samples into categories as Responder and Non-responder [37]. One of the methods to assess the performance of a classificator is the ROC analysis. Based on transcriptomic data and clinical information from a large dataset treated with ICBs therapies (http://rocplot.org/immune), we build MTHFR expression in pretreatment and ontreatment tumor specimens as the training set. We separately analyzed the ROC of MTHFR in all ICBs, Anti PD-1 (CD279) therapy (nivolumab, pembrolizumab), Anti PD-L1 (CD274) therapy (atezolizumab, durvalumab), Anti CTLA4 (CD152) therapy (ipilimumab) groups. The Mann–Whitney U test is a rank-based non-parametric test. Characteristics of the groups are presented by employing a box-and-whisker plot.

### Verification the expression of MTHFR by immunohistochemistry (IHC)

The Human Protein Atlas was used to verification the expression of MTHFR in cancer tissue samples by IHC. Two antibodies HPA076180 and HPA077255 were used to detect the expression of MTHFR in cancer tissues. In the Human Protein Atlas, IHC-based images of normal and cancer tissues are presented. Antibodies are labeled with DAB (3,3'-diaminobenzidine) and the brown staining indicates where an antibody has bound to its corresponding antigen. We examined the immunostaining of 144 tissues from 44 different normal tissue types using tissue microarrays, and a total of 216 cancer samples representing 20 different types of cancer. Each sample is represented by 1 mm tissue cores.

## Results

### Expression of MTHFR across tissues, cancers and pathological stages

**Fig. 1:**
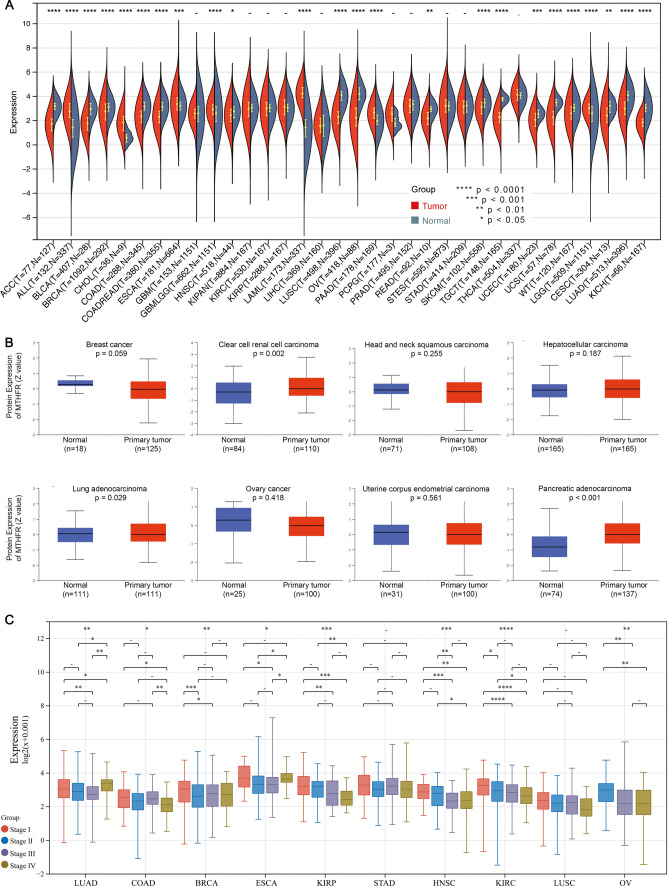
Expression of *MTHFR* across tissues, cancers and pathological stages. **A** Expression of MTHFR between tumor tissues from TCGA database and normal tissues from TCGA and GTEx database. **B** Protein expression level of MTHFR was analyzed between normal tissue and primary tissue of eight tumors. **C** The expression levels of the *MTHFR* gene were analyzed by the main pathological stages of 8 cancers. Log2 (TPM + 1) was applied for log-scale.. **** p < 0.0001, *** p < 0.001, ** p < 0.01, * p < 0.05. All p-values were adjusted

We firstly analyze the expression level of gene and protein in cancer to show whether the MTHFR could influence the mechanism of cancer. Expression alterations play important roles in cell function and regulating up and down genes’ transcriptome features. Overall, MTHFR gene expresses significantly different in 24 cancers types. MTHFR gene shows significantly lower expression in 17 tumors, including BRCA, CESC, LUAD, et al. However, the expression level of MTHFR gene has higher expression in 7 tumor samples (Fig. 1A) and exhibited widely expression in normal tissues, such as ALL, GBML, LAML, et al. Specifically, MTHFR shows highest expression in the LAML, lowest expression in the LIHC (Fig. 1A). According to the CPTAC datasets, the total protein level of MTHFR is significantly higher in KIRC and PAAD (Fig. 1B), lower in LUAD. The “Pathological Stage Plot” analysis shows lower MTHFR expression is significantly correlated with the higher pathological stages in 8 cancers, including BRCA, COAD, LUAD, ESCA, KIRC, KIRP, HNSC, OV. Groups comparison also demonstrates the relationship significantly.

### Prognostic significance of MTHFR across cancer types

**Fig. 2:**
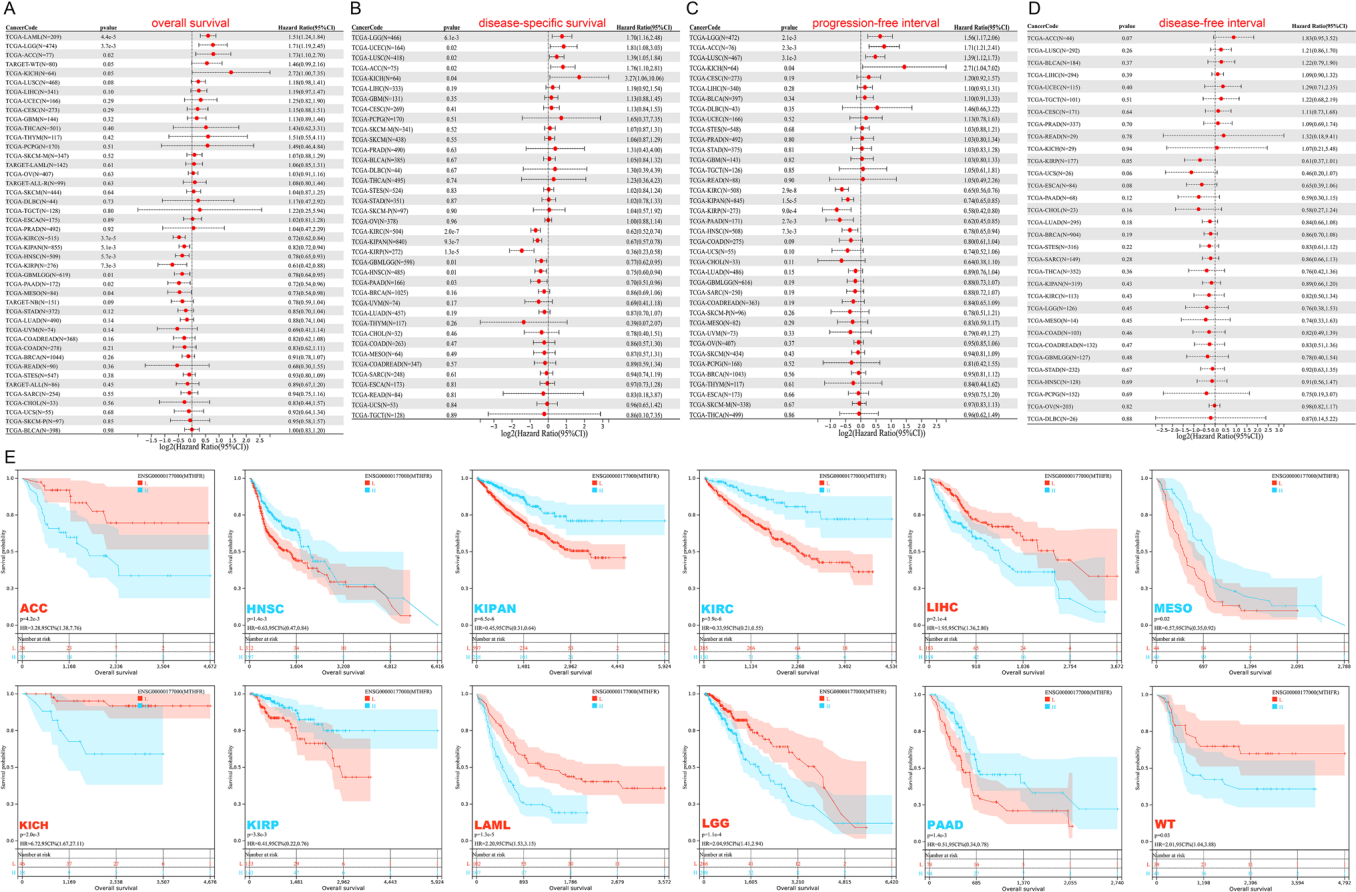
Prognostic significance of MTHFR across cancer types. **A** Forest map showing the univariate cox regression results of MTHFR for overall survival of 44 tumors. **B** disease-free interval of 32 tumors. **C** disease-specific survival of 38 tumors. **D** progression free survival of 38 tumors. **E** Kaplan–Meier survival plot of patients grouped by low and high expression of MTHFR for overall survival in cancer types. Centre line of boxplot is the HR, and bounds of box are the 95% confidence levels

Tumor samples are divided into high and low expression groups depending on the expression levels of MTHFR. The correlations between MTHFR expression and prognostic significance in different types of cancer are examined. In Fig. 2A, high expressed MTHFR is linked to better prognosis of OS in LGG, LAML, ACC, but associated with the poorer OS prognosis in 7 cancer types, including KIPAN, HNSC, PAAD, et al. According to DSS analysis data (Fig. 2B), high MTHFR expression is predictor of favorable DSS among patients with LGG, UCEC, LUSC, et al., but go against DSS in KIRC, KIRP, GBMLGG, HNSC, PAAD. Additionally, high expression of the MTHFR is related to better PFI in LGG, LUSC, ACC, KICH (Fig. 2C). In contrast, low MTHFR expression could predict the favorable PFI in KIRC, KIRP, HNSC, PAAD. Expression of MTHFR of all cancer types shows no relationship to DFI (Fig. 2D).

Using the Kaplan–Meier plotter tool, analysis of the survival probability of OS presents a significant correlation between low expression of MTHFR and favorable OS prognosis in ACC, LIHC, LAML, LGG, KICH, WT. Meanwhile, high expressed MTHFR could predict the OS among patients with KIRC, HNSC, PAAD, MESO (Fig. 2E).

### Alteration of MTHFR gene across tumor types

**Fig. 3:**
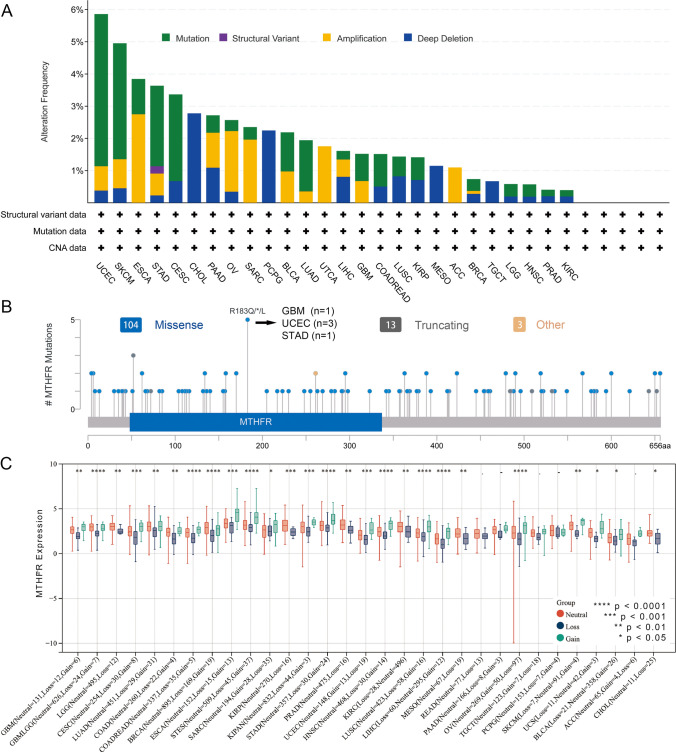
Alterations of MTHFR gene across tumor types.** A** 4 different types of alterations of MTHFR gene, including mutation, structure variant, amplification, and deep deletion are shown totally different across tumor types using the cBioPortal tool. **B** Type, site, and case number of the MTHFR genetic alterations. **C** Differences in MTHFR expression between Neutral, loss and gain in 26 tumors. Centre line of boxplot is the median, and bounds of box are the upper and lower quartile. **** p < 0.0001, *** p < 0.001, ** p < 0.01, * p < 0.05

Gene alterations of MTHFR are totally different across tumor types (Fig. 3A). MTHFR alteration is most prevalent in UCEC with mutation (4.73%, 25 cases), Amplification (0.76%, 4 cases) and Deep Deletion (0.38%, 2 cases). The amplification type of copy-number alterations (CNA) is the primary type in the ESCA and Uterine Carcinosarcoma cases, which shows an alteration frequency of 2.75% (5 cases) and 1.75% (1 case) respectively. It is worth noting that all Cholangiocarcinoma (2.78%), Pheochromocytoma & Paraganglioma (2.25%), Mesothelioma (1.15%) and Testicular Germ Cell (0.67%) Tumors have copy number deletion of MTHFR (Fig. 3A). Further details are provided in Fig. 3B, including the type, site, and case number of the MTHFR alterations. MTHFR missense mutations are the most prevalent alteration. In 3 cases of UCEC, 1 case of GBM and STAD, R183Q/*/L alteration in the domain is able to induce a missense mutation of the MTHFR gene (Fig. 3B). An alteration to MTHFR protein occurs when R (Arginine) is converted to Q (Glutamine)/L (Leucine) at the 183 sites. Furthermore, we observe the significant differences in MTHFR expression between Neutral, loss and gain in 26 tumors. As shown in Fig. 3C, gain mutation of MTHFR shows highest and lowest expression in the ESCA and BLCA respectively, loss mutation of MTHFR shows highest and lowest expression in the ESCA and LIHC respectively.

### Machine learning identifies stemness features associated with MTHFR

**Fig. 4:**
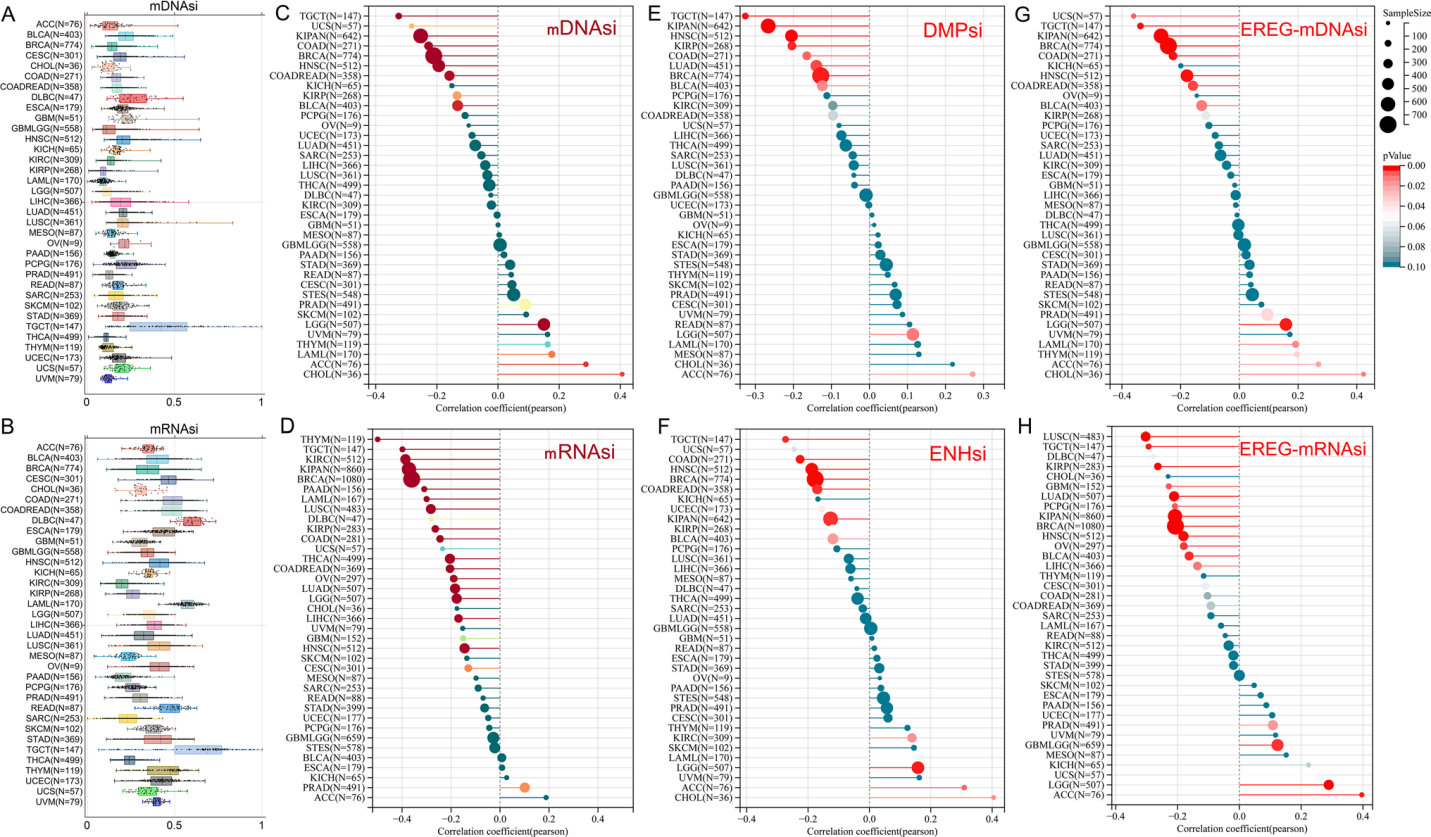
Machine Learning Identifies Stemness Features Associated with MTHFR across cancer types. **A** Features of mDNAsi in cancers. **B** Features of mRNAsi in cancers. **C**–**H** The expression of MTHFR was negatively correlated with the mDNAsi, mRNAsi, DMPsi, ENHsi, EREG-mDNAsi and EREG-mRNAsi across cancer types

Because of related with oncogenic dedifferentiation across tumor types according to previous study [38], machine learning identifies mDNAsi (Fig. 4A) and mRNAsi (Fig. 4B) stemness indices of MTHFR that are Scales ranged from 0 (low) to 1 (high) are analyzed to show the initiation and evolution of cancer. A correlation between cancer stemness index and ICBs response may lead to the discovery of potential therapeutic targets [38]. Using DNA methylation levels, the mDNAsi reflects epigenetic stemness characteristics, while using mRNA expression levels mRNAsi reflects the transcriptomic stemness characteristics. As we can see from Fig. 4C–H, the expression of MTHFR could predict the stemness indices of mDNAsi, mRNAsi, DMPsi, ENHsi, EREG-mDNAsi and EREG-mRNAsi in the most cancers. Specifically, we calculated Pearson correlation in 37 tumors. Results suggests that mDNAsi is significantly negatively correlated with MTHFR in 9 tumors, such as COAD, COADREAD, BRCA, et al. Meanwhile, Correlation of mRNAsi and MTHFR shows negative in 18 tumors. High expression of MTHFR predict low DMPsi significantly among patients with LUAD, COAD, BRCA, KIRP, KIPAN, HNSC, TGCT, BLCA. ENHsi is significantly negatively correlated with MTHFR in COAD, COADREAD, BRCA, et al. EREG-mRNAsi is significantly negatively related with MTHFR in 12 tumors. EREG-mDNAsi is significantly negatively correlated with MTHFR in COAD, COADREAD, BRCA, et al.

### Immune infiltration analysis of MTHFR in cancer

**Fig. 5:**
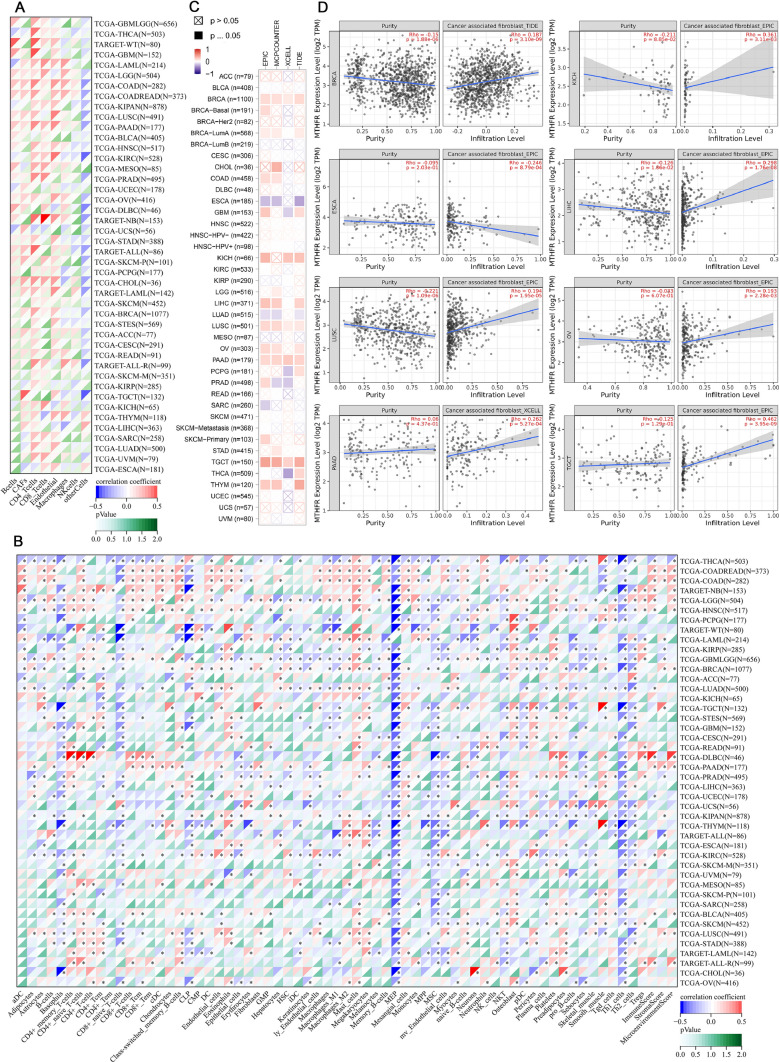
Immune infiltration analysis of MTHFR in cancer. **A** The correlation between MTHFR and infiltration level immune cells using EPIC algorithm. **B** The correlation between 67 types of immune cell infiltration scores of 44 tumor type and MTHFR using XCELL algorithm. **C **Correlation analysis between *MTHFR* expression and immune infiltration of CAFs in different algorithms. **D** The scatter plot shows positive correlation between MTHFR expression and the estimated infiltration value of CAFs in 8 types of tumors

Tumor-infiltrating immune cells, as prominent components of the TME, are closely linked to the tumorigenesis, progression and metastasis. In EPIC algorithm (Fig. 5A), MTHFR is positively correlated with B-cells, cancer-associated fibroblasts (CAFs), CD4+T-cells, CD8+T-cells in almost tumors. After a series of analyses by XCELL we obtained 67 types of immune cell infiltration scores of 44 tumor types from a total of 10179 tumor samples (Fig. 5B). Subsequently we calculated the Pearson’s correlation coefficient between MTHFR and immune cell infiltration score in each tumor using the corr. test function of R software package psych to determine the significantly related immune infiltration score. Finally, the MTHFR expression showed significantly correlated with immune infiltration in all 44 cancer types. In XCELL algorithm of most cancers, MTHFR is positively correlated with the, CD4+T-cells, CD8+T cell, CD4 naive T cell, mast cell and osteoblast and others, negatively correlated with CD8 naive T cell, Th1 and Th2 cell, Mucoid Exopolysaccharide, et al. But the results are quite different when it comes to a specific tumor. These phenomena could be a clue to further research about the tumorigenesis and immunotherapy of MTHFR.

Moreover, CAFs in the stroma of the TME were reported to participate in modulating the function of various tumor-infiltrating immune cells. Herein, using the XCELL, MCPCOUNTER, EPIC and TIDE algorithms, we investigated the possibility of a relationship between the level of infiltration of different immune cells and the expression of the MTHFR gene among TCGA cancer types (Fig. 5C). A statistically positive correlation between MTHFR expression and the estimated infiltration value of CAFs is observed in 8 types of tumors, including BRCA, LIHC, LUSC, et al. but negative correlation in ESCA and LUAD. The scatter plot data of the above tumors processed by one algorithm are presented in Fig. 5D. Except for the negative relationship to ESCA, the MTHFR expression is positively correlated with the CAFs infiltration in the other 7 tumors.

### Enrichment analysis of MTHFR across cancer types

**Fig. 6:**
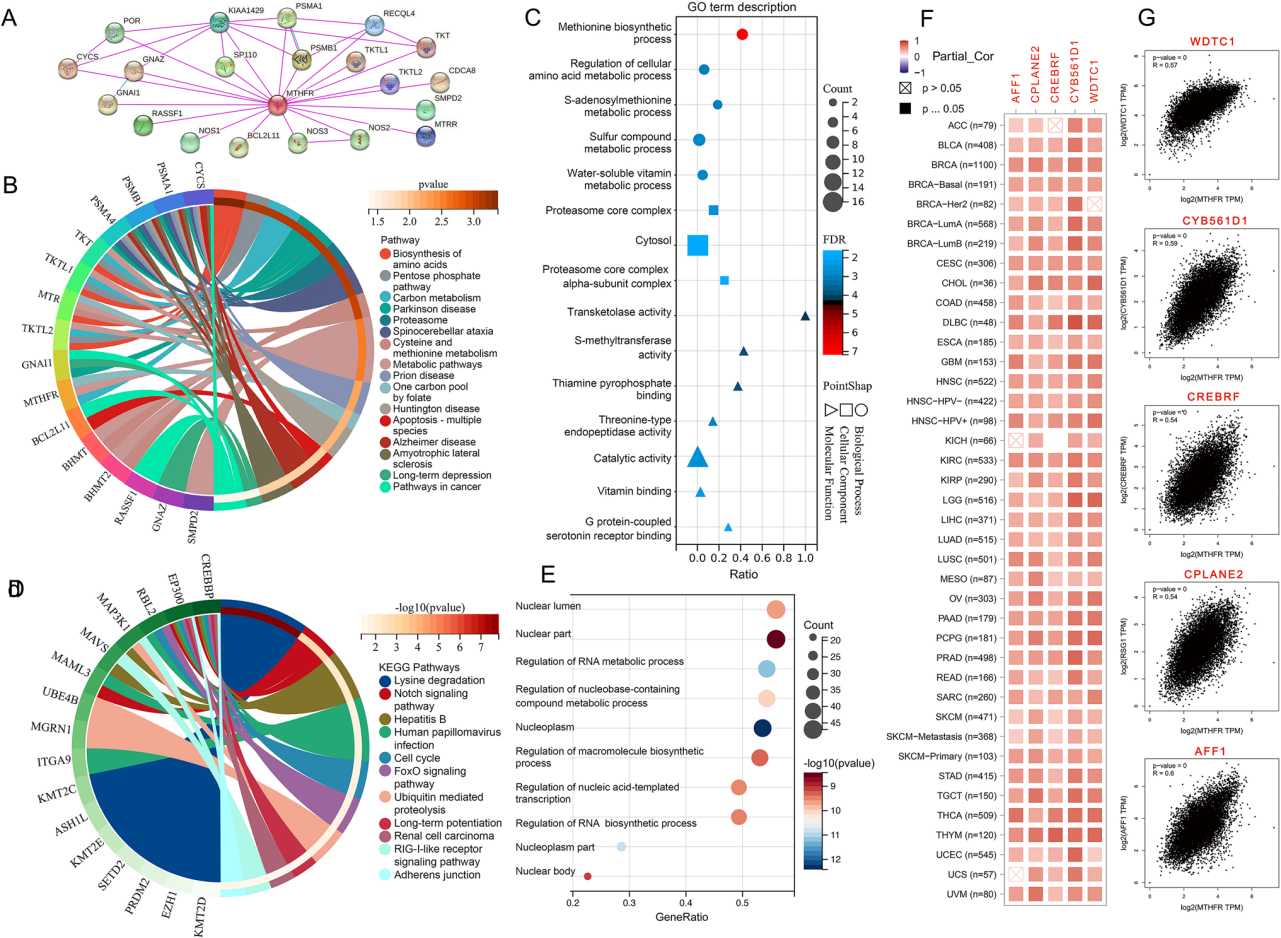
Enrichment analysis of MTHFR across cancer types**. A** Interaction network of 20 MTHFR-binding proteins using the STRING tool. **B** KEGG pathway analysis of the MTHFR-binding protein. **C** GO enrichment analysis of the MTHFR-binding protein. **D** KEGG pathway analysis of the MTHFR most correlated genes. **E** GO enrichment analysis of the MTHFR most correlated genes. **F** The corresponding correlation of 5 genes and MTHFR in the detailed cancer types using the GEPIA2 heat map. **G** Scatter plot of 5 genes most correlated with MTHFR expression in TCGA projects

To Study the molecular mechanism by which the MTHFR gene contributes to tumorigenesis, we conducted GO and KEGG Pathway enrichment analyses to identify the targeting of the MTHFR expression-correlated genes and MTHFR-binding proteins. by using the STRING tool, 20 MTHFR-binding proteins are MTRR, KIAA1429, PSMA1, PMSB1, NOS1/2/3 and others (Fig. 6A). The KEGG data of MTHFR-binding proteins suggests that MTHFR is most related with Biosynthesis of amino acids, Pentose phosphate pathway, Carbon metabolism, Parkinson disease, et al. (Fig. 6B). According to the GO enrichment analysis of Biological Process (BP), Cellular Component (CC) and Molecular Function (MF) data, MTHFR is most related with Methionine biosynthetic process of BP, Proteasome core complex of CC and Transketolase activity of MF (Fig. 6C).

Utilizing the GEPIA2 tool, we combined all TCGA tumor expression data to identify the top 100 genes associated with MTHFR expression. We combined the genes datasets to perform GO and KEGG enrichment analysis. The KEGG data suggests the most related 11 pathways, including Lysine degradation, the NOTCH signal pathway, Hepatitis B and Human papillomavirus which could be influenced by MTHFR in tumor pathogenesis (Fig. 6D).The GO enrichment analysis further indicates that MTHFR plays an important role in nuclear lumen, nuclear part, regulation of RNA metabolic process, regulation of nucleobase-containing compound metabolic process, nucleoplasm and others (Fig. 6E). In Fig. 6F, the MTHFR is most positively correlated with AFF1 (ALF transcription elongation factor 1, R = 0.60), CPLANE2 (ciliogenesis and planar polarity effector complex subunit 2, R = 0.54), CREBRF (CREB3 regulatory factor, R = 0.54), CYB561D1 (cytochrome b561 family member D1, R = 0.59), WDTC1 (WD and tetratricopeptide repeats 1, R = 0.57). Additionally, the corresponding scatter diagrams indicate positive correlations between MTHFR and the above five genes separately (Fig. 6G).

### Heterogeneity and MTHFR

**Fig. 7:**
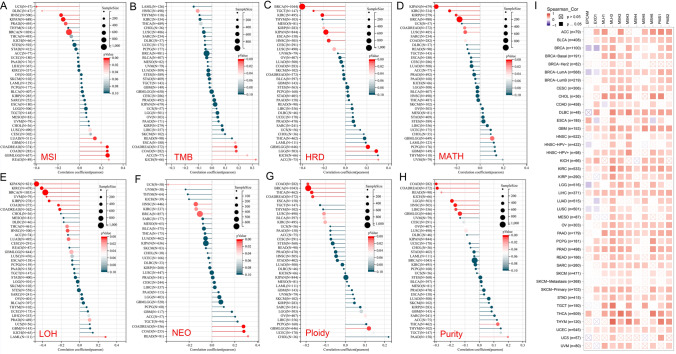
Heterogeneity and MTHFR. **A**–**H** MTHFR shows significantly positive correlation with the expressions of MSI, TMB, HRD, MATH, LOH, NEO, Ploidy and Purity of Simple Nucleotide Variation in cancers. **I** Relationship between MTHFR and 9 MMR genes (MLH1, PMS2, MSH2, MSH6, EPCAM, MLH3, PMS1, EXO1) in different tumors

Our research demonstrates MTHFR expression is a strong predictor of heterogeneity after analyzing the relations with biomarkers MSI, TMB, HRD, MATH, LOH, NEO, Ploidy and Purity. Firstly, MTHFR predicts significantly positive relationship to Gene expression Data of MSI [39] in COAD, COADREAD, LUAD, et al., and negative relationship in BRCA, KIPAN, PRAD, et al. (Fig. 7A). We observed MTHFR significantly positively correlated with TMB in COAD, COADREAD, ESCA, ACC and KICH, negatively correlated in HNSC. Accurate detection of HRD [40] is clinical relevance as it is indicative of sensitivity to targeted therapy with poly ADP ribose polymerase inhibitors (PARPi) as well as to DNA damaging reagents [41]. HRD shows significantly positively correlated with MTHFR in ACC, GBMLGG, LGG, but negatively correlated with MTHFR in CESC, BRCA, ESCA, et al. (Fig. 7C). MATH is a novel, non-biased, quantitative measure to assess intra-tumor heterogeneity based on next-generation sequencing data. In our study, the MATH shows significantly positively correlated with MTHFR only in GBMLGG. in contrast, MATH is negatively correlated with MTHFR in BRCA, COADREAD, KIPAN, and LUSC (Fig. 7D). Correlations between MTHFR and LOH, NEO, Ploidy and Purity are also significant in specific tumors (Fig. 7E–H).

Different detection methods are used for evaluating the same biological effect, such as DNA for determining MSI status or IHC for detecting MMR protein expression [42]. Nine MMR genes, such as MLH1, PMS2, MSH2, MSH6, EPCAM, MLH3, PMS1, EXO1, have been found in the human MMR system [43]. Low expression of MMR genes meaning less protein will be detected by IHC may be seen as negative or dMMR. In Fig. 7I, MTHFR is significantly positively correlated with most MMR genes in most cancer types. Generally, Mutated MTHFR expressing low level of protein may be correlated with dMMR in IHC. This phenomenon need be verified in future research.

### MTHFR and IRGs across cancer types

**Fig. 8:**
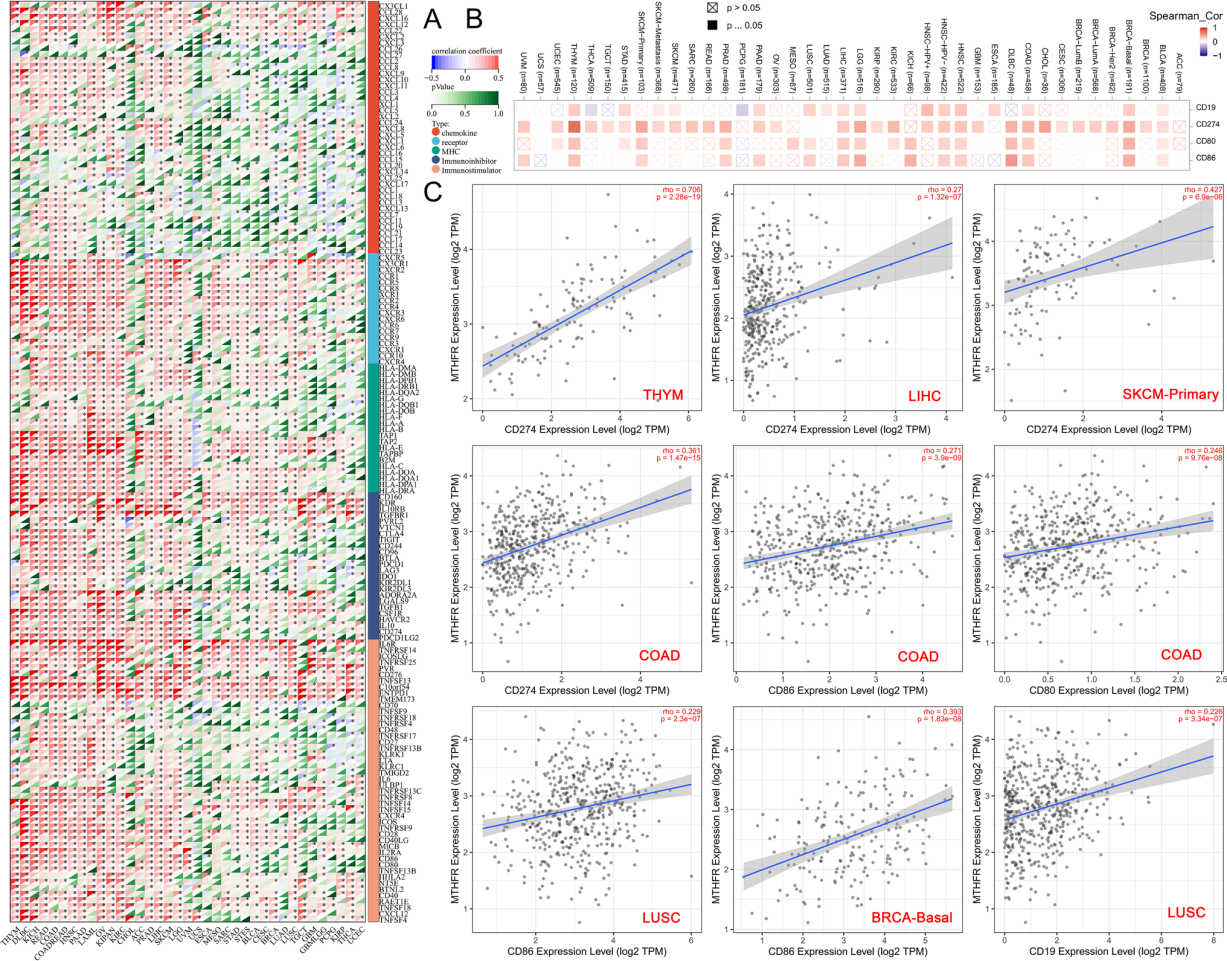
MTHFR and immune related genes across cancer types. **A** MTHFR is positively correlated with the most gene expression of immunoinhibitors, immunostimulators, MHC molecules, chemokines and their receptors in most cancers (* P < 0.05). **B** Heat map displays the positve correlation between CD19, CD274, CD80/CD86 and MTHFR in most cancers. **C** Scatter plot shows the significant correlation between the immune relate genes and MTHFR in specific cancers

Our analyses aim to depict the immunological role of MTHFR which is critical in determining the types of cancers benefit from immunotherapy. Correlation analyses demonstrate that the MTHFR is positively correlated with the most expression of immunoinhibitors, immunostimulators, major histocompatibility complex (MHC), chemokines and their receptors in most tumors (Fig. 8A). MTHFR demonstrates merely positive relations to dozens of IRGs, such as CX3CR1, CCR4, TAP2, HLA-E, TGFBR1, IL6R, TNFSF13, et al. However, heat map displays several cancer types such as CHOL and UCS show almost few relationships between MTHFR and the immune related genes. Based on ICBs, We select the somatic genes CD19, CD274, CD80, CD86 as the most representative genes for analysis. The results demonstrate that MTHFR is significantly positively correlated with CD80/CD86 in BRCA − Basal, COAD, HNSC − HPV ( −), KICH, LIHC, LUSC, PRAD and SKCM − Primary, with CD274 in COAD, HNSC, LIHC, SKCM and others, with CD19 in LUSC, BRCA − Basal, COAD and others. (Fig. 8B). To be specific, MTHFR is significantly correlated with CD274 in THYM (P = 2.38e−19), LIHC (P = 1.32e−07), COAD (P = 1.47e−15), with CD86 in COAD (P = 3.9e−09), in LUSC (P = 2.3e−07), in BRCA (P = 1.83e−08), with CD80 in COAD (P = 9.76e−08), with CD19 in LUSC (P = 3.34e−07), et al. (Fig. 8C).

### MTHFR act as a potential biomarker of ICBs

**Fig. 9:**
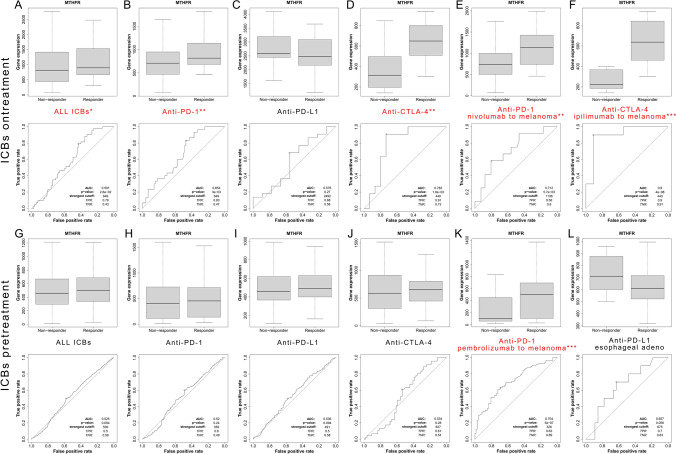
MTHFR act as a potential biomarker of ICBs. **A**-**F** Response and no response group of ontreatment patients show different expression of MTHFR and ROC plotter for all ICBs, anti-PD-1, anti-PD-L1, anti-CTLA-4, ipilimumab therapy (melanoma) and nivolumab therapy (melanoma). **G**-**L** Response and no response group of pretreatment patients show different expression of MTHFR and ROC plotter for all ICBs, anti-PD-1, anti-PD-L1, anti-CTLA-4, pembrolizumab therapy (melanoma) and anti-PD-L1 (esophageal adenocarcinoma). Log2 (TPM+1) was applied for log-scale.. *** p < 0.001, ** p < 0.01, * P < 0.05

Area under curve (AUC) shows how well the test separates the responder and non-responder groups. The larger the area under the ROC curve, the more useful is the measurement to predict treatment response. After calculating data of Ontreatment patients (Fig. 9A–F), MTHFR shows significant relationship with the response to all ICBs therapy (AUC = 0.591, P = 0.028) and anti-PD-1 therapy (AUC = 0.654, P = 0.003). MTHFR could act as a biomarker with potential clinical utility for anti-CTLA-4 therapy (AUC = 0.782, P = 0.002). Precisely, MTHFR could act as blockbuster biomarker for ipilimumab to melanoma of anti-CTLA-4 ontreatment (AUC = 0.9, P < 0.0001) and potential cancer biomarker for nivolumab to melanoma of anti-PD-1 ontreatment (AUC = 0.712, P = 0.006). When coming to the data of pretreatment patients (Fig. 9G–L), MTHFR could act as a potential biomarker for pembrolizumab to melanoma of anti-PD-1 therapy (AUC = 0.704, P < 0.0001).

### Verification the expression of MTHFR by IHC

**Fig. 10:**
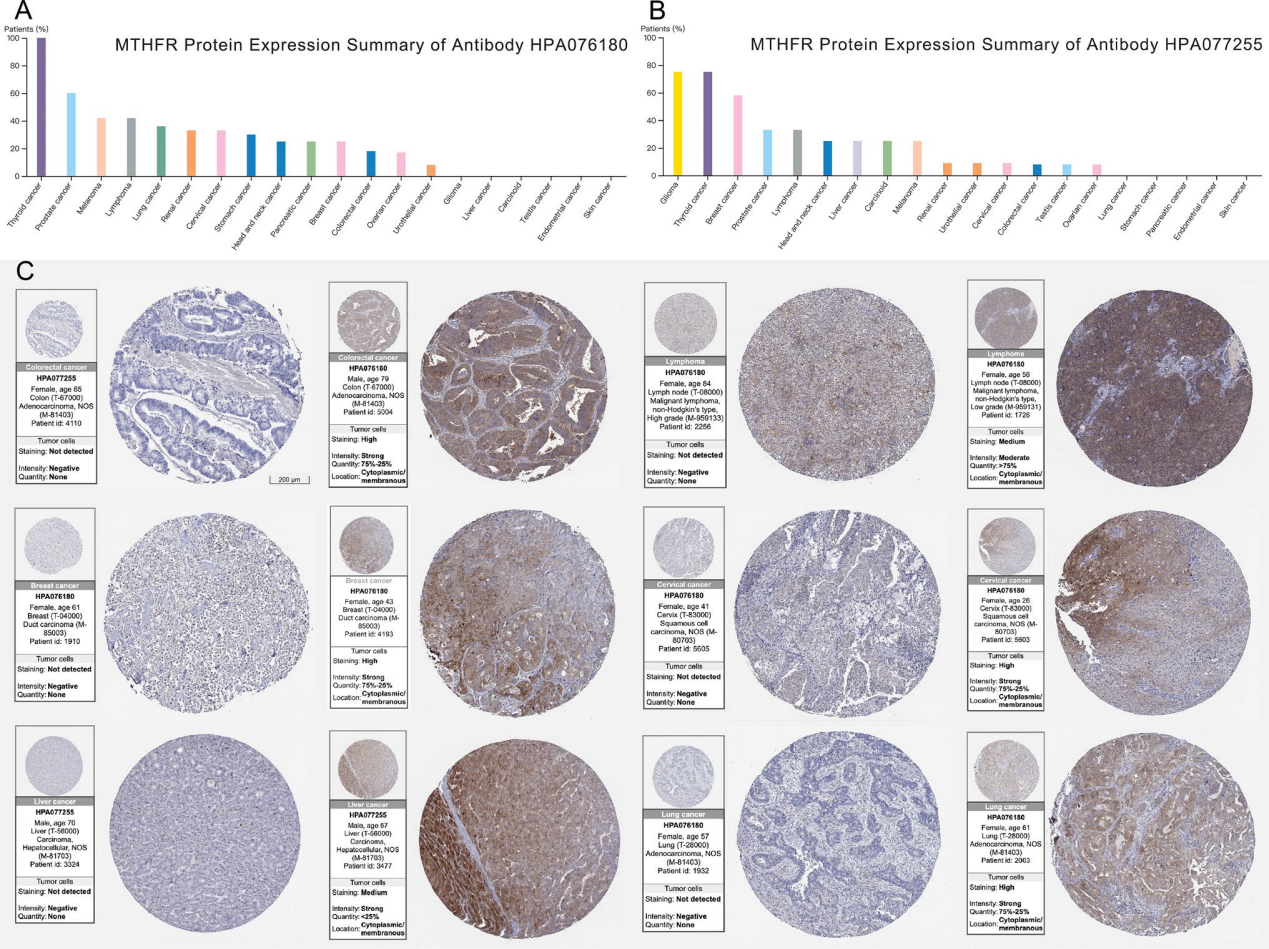
Verification of the expression of MTHFR by immunohistochemistry. **A**, **B** MTHFR protein expression summaries of antibodies HPA076180 and HPA077255 separately in different cancer tissues. **C** Positive and negative control of IHC in 6 cancers, including COAD, malignant lymphoma (non-Hodgkin’s type), breast duct carcinoma, cervix squamous cell carcinoma, LIHC and LUAD

For better clinic application of MTHFR to assist treatment and research, we verified the IHC across cancer types. Figure 10A, B indicates the MTHFR IHC summaries of antibodies HPA076180 and HPA077255 separately in different cancer tissues. Most cancers show the strong and medium positive expression of MTHFR, including thyroid cancer, lung cancer, breast cancer, et al. But the two kinds of antibody are not able to achieve positive results for all cancers. HPA076180 shows no positive IHC staining in 6 cancers, including Glioma, liver cancer, carcinoid, testis cancer, endometrial cancer and skin cancer patients. HPA077255 shows no positive IHC staining in 5 cancers, including lung cancer, stomach cancer, pancreatic cancer, endometrial cancer and skin cancer. Researchers designing experiment should take a note of this. Figure 10C shows the IHC staining images of positive and negative control of 6 prevalent cancers typically, including colon adenocarcinoma, malignant lymphoma (non-Hodgkin’s type), breast duct carcinoma, cervix squamous cell carcinoma, liver carcinoma hepatocellular and lung adenocarcinoma.

## Discussion

The emergence of evidence has revealed the MTHFR could act a potential biomarker of the initiation, prognostic significance, TME, immune infiltration and ICBs across different tumor types. Among 24 different cancers, we observe significant differences in the expression of MTHFR between tumor and normal samples. 7 tumors exhibited higher expression and 17 tumors exhibited lower expression, indicating that MTHFR may serve as a protective factor against most tumors. Previous studies indicated that MTHFR was lower expressed in BRCA tissues [17, 44] compared with normal tissues, which is consistent with those we obtained. Subsequently, MTHFR protein functions study indicates a higher expression level of MTHFR total protein in the KIRC, LUAD and PAAD compared with that of normal controls, indicating the MTHFR protein could affect the susceptibility of tumors. That MTHFR expressions is significantly negatively associated with Pathological stages in 8 tumors also suggests the potential protective role of MTHFR against the development of tumor.

Tumor Growing and metastasis must based on the DNA synthesis. MTHFR catalyzed conversion of 5,10-CH2-THF to 5-methyl-THF is irreversible in cells at the expense of 5,10-CH2-THF and 10-formyl-THF, both of which are crucial to DNA synthesis [45]. Downregulation of MTHFR mRNA and protein means less 5-methyl-THF produce, more 5,10-CH2-THF and 10-formyl-THF could be used to synthesize DNA and tNRA [46]. On the other side, high level of homocysteine caused by low level of 5-methyl-THF which is due to downregulation of MTHFR may metabolized into glutathione [45]. Research demonstrated that high level of glutathione is related with tumors [47].

Furthermore, the survival prognosis analysis for the MTHFR gene suggests different tumor produces different result. Compared with 3 tumors of high expression, 7 tumors of low expression predict the favorable OS prognosis in this study, suggesting the MTHFR is complex in prognoses. But the phenomena cannot be shown in most cancers. For the digestive system, high MTHFR expression exhibits poor survival in LIHC.

As no previous study has reported the correlation relationship between MTHFR expression and the tumor stemness indices across cancer types, so we identified MTHFR is negative correlation to the stemness index in most tumors and could be a potential therapeutic targets predicting the response to ICBs [38]. MTHFR may act as suppressor of some tumors. Distinct evidence was merited for the potential role of expression of MTHFR in the tumorigenesis. Our results also demonstrate that MTHFR undergoing alteration could acquire oncogenic properties and enhance the stem-like characteristics of cancer cells, suggesting that alteration of MTHFR has major effect on the regulation of the stemness of cancer stem cells. Higher mRNAsi is associated with basal, Her2 and luminal B subtypes that are more aggressive than the hormone-dependent luminal A group [32]. The negative correlation between stemness indices and outcome for some tumor types may indicate malignant cell origins or the impact of TME. Importantly, higher stemness index was associated with poorer prognosis and greater oncogenic dedifferentiation reflected by histological grade [38]. High mDNAsi was strongly associated with high grade, poor OS and PFS of glioblastomas. In adrenocortical carcinoma, higher mRNAsi was associated with shorter OS and greater metastatic potential [48]. We could conclude that the lower expression of MTHFR, the higher the tumor stemness index, the stronger the activity of tumor stem cells, and the lower the degree of tumor differentiation [38]. Hypothesis emerges that patients with MTHFR dysfunctional mutation taking supplement to enhance the metabolism route may decrease the stemness indices and improve the prognosis need more research.

Gene expression epigenetic regulation is further complicated by enhancer regulatory regions, which affected by specific epigenomic changes Based on genome-wide DNA methylation assays and histone mark annotations. Ordonez R summarized enhancer methylation regulating the DNA molecular and biological function contributed to the fine-tuning of gene expression [49]. In fact, active enhancers display lower levels of DNA methylation, so subsequent transcription factor binding and activating transcription is related with hypomethylation of enhancer DNA [49]. In mature individuals, methylation of enhancer DNA plays a role in T-cell lineage specification and granulopoiesis as well as terminal differentiation processes [50]. A malignant phenotype causing cellular de-differentiation is caused by abnormal enhancer methylation in both solid and hematological tumor cells [51]. In our results, the specific relationship between MTHFR and enhancer DNA methylation is worth to be investigated in the early transforming process and the malignant phenotype maintenance. Especially COAD and BRCA are observed to be negatively correlated with ENHsi and other 5 types of stemness indices, suggesting that MTHFR may function as a protective factor to these tumors. In epigenetic research, stemness indices are a promising new topic. A significant amount of progress in annotation and identification of relationship between MTHFR and stemness indices is expected in the coming future.

Cancer initiation, progression, and metastasis are closely related to tumor-infiltrating immune cells in the TME [52]. Applying XCELL, we gain insight into the TME and composition of immune cell infiltrates. Our result systematically suggested negative correlation between MTHFR and the naive T cell, Th1 and Th2 cell, CD8 + naive T cell in most cancers based on XCELL algorithms. Th1 cell plays a key role in mounting a host defense against intracellular pathogens. Pathologic complete response at surgical resection was associated with a multimarker score containing higher levels of Th1 cells and CD8+central memory T-cells (CD8 + TCM) [53]. Polarized Th1 cytokine microenvironment induced by IFNα with CTLA4 blockade can enhance cytotoxicity of the antitumor CD8 + T cell. So, because of negative correlating with Th1 in most tumors, MTHFR could act as a suppressor of the microenvironment of th1 and to influence the host defense and antitumor effect. In the TME, various tumor-infiltrating immune cells are reported to be stimulated by CAFs [54]. We found CAFs infiltration had the significantly positive correlation with MTHFR in BRCA, KICH, LIHC, LUSC, OV, PAAD and TGCT, suggesting MTHFR may influence CAFs to modulate tumor immunity.

It is very interesting when it comes to the KEGG enrichment analyses of MTHFR expression-related genes. We identified the potential impact of “Human papillomavirus infection” is significant pathway associated with MTHFR in the pathogenesis of cancers. According to these data, MTHFR may play key roles in the associated cancer development and occurrence. A study based on 13 case–control studies demonstrates a significantly increased cervical cancer risk with MTHFR A1298C polymorphism, but failed to obtain data of HPV-infection status subgroup [55]. It would be hint for more mechanism research that MTHFR A1298C is more likely to be a potential target of treatment for HPV-cervical cancer.

In recent years, cancer immunotherapy (CIT) has become a new treatment option, although only a small percentage of patients experience clinical improvements. Thus, it is imperative to identify predictive biomarkers. CIT response has been predicted by several ICBs genes, including PDCD-1, CD274 and CTLA-4 [56]. According to our analysis, we observed significant correlations between the MTHFR gene and CD274, CD80/CD86, CTLA4. These findings give a hint to us that the expression of MTHFR may affect the response of ICBs. The overexpression pattern of MTHFR is TME specific in some tumors, which demonstrates the potential of MTHFR as a biomarker for normalized CIT. The positive correlation between MTHFR and CD274 in COAD, suggesting that COAD with higher expressed MTHFR may be a suitable candidate cancer type for PD-L1 therapy.

Along with TMB, It is possible that MSI status is one of the most reliable predictors of ICBs therapy [57]. In a proof-of-concept study, MSI status was correlated with response to pembrolizumab in 87 patients with 12 cancer types [58]. MSI predicting pembrolizumab response led to the FDA's approval of the first tumor-agnostic drug in May 2017. Patients with MSI-high (MSI-H) responded favorably to nivolumab and MEDI0680 for anti-PD-1 treatment, durvalumab for anti-PD-L1 treatment, and ipilimumab for anti-CTLA-4 treatment [59, 60]

Our study may be the first one to present evidence of the potential correlation between MTHFR expression and MSI or TMB across tumor types. That both TMB and MSI showed significantly negative correlation to MTHFR in HNSC, positive correlation to COADREAD inspire us that MTHFR could be investigated as a possible predictor along with MSI and TMB for immune signatures for these tumors. Positive correlation between MTHFR and the MSI, TMB and the MMR genes in COAD totally indicates MTHFR could be a biomarker of ICBs. For the lower MTHFR expression patients, whether the drugs that are supplement to the metabolism of MTHFR could integrate the pathways and assist the response of CIT could be further investigated.

Evidence showed intratumor heterogeneity has a negative impact on the clinical therapeutic effect of immunotherapy [61]. MATH, a practical and useful way to measure intratumor heterogeneity, may serve as a significant biomarker for the prognosis of patients. MATH score demonstrates positively correlated with heterogeneity and negatively correlated with prognosis in HNSC [62], BRCA [63], READ [64], and LUAD [65], UCEC [66]. In our results, significantly negative correlation between MATH and MTHFR in BRCA, COADREAD, KIRC, KIRP and LUSC demonstrated that tumors with lower expression of MTHFR may show higher heterogeneity and be predictor of CIT, suggesting that MTHFR is a potential target for the development of combination therapy.

The homologous recombination pathway plays a critical role in the high-fidelity repair of DNA double strand breaks (DSBs) and involves a number of genes, including BRCA1 and BRCA2. Inactivation of such genes leads to HRD, which results in increased levels of genomic changes [67]. HRD was found in 6% of metastatic cancers, 30% in ovarian cancer and breast cancer, prostate cancer and pancreatic cancer (12% ~ 13%) [41]. It is of clinical importance to detect HRD accurately, as it indicates sensitivity to targeted therapies like poly ADP ribose polymerases inhibitors (PARPi) [68] and DNA damaging agents [67]. Recent study supported Adding maintenance olaparib to bevacizumab after first-line chemotherapy provided significant improvement in progression-free survival in advanced ovarian cancer patients with HRD-positive tumors, including those without a BRCA mutation [69]. In our study, MTHFR negatively correlated with HRD in 9 tumors suggests low expression of MTHFR could reduce the high-fidelity repair of DSBs, increase genomic changes, indicate sensitivity to targeted therapies in these tumors.

There are few researches directly on the relationship between MTHFR and ICBs across cancer types. So, our research could be the first one concentrating on the efficacy of MTHFR and ICBs. From the Analysis of MTHFR expression in pre-treatment and on-treatment tumor specimens as the training set, we validate the expression of MTHFR is significantly related with the response of ICBs. Surprisingly we found higher expression of MTHFR could receive more response to total ICB therapy, anti-PD-1 therapy and anti-CTLA-4 therapy. Precisely, MTHFR could act as blockbuster biomarker for ipilimumab therapy (AUC = 0.9) and top-quality cancer biomarker for nivolumab therapy (AUC = 0.712) to melanoma patients. When coming to the data of pretreatment patients, MTHFR could act as a top biomarker for pembrolizumab therapy (AUC = 0.704) to melanoma patients. The results hint the higher expression of MTHFR the more response to the ICB therapy. MTHFR could act as a potential biomarker of ICBs. But the defect of the research is the sample size of ipilimumab therapy to melanoma is small, only including 21 patients in total, non-responder and responder are 11 and 10 separately. Subsequently we will conduct further research to investigate the correlation, mechanism and metabolism between MTHFR and ICBs in cancers.

IHC staining patterns may reflect tumor heterogeneity and interindividual differences in diverse expression of proteins. IHC test could be an easy way to verify relationship between MTHFR with MMR genes and PD-L1 predicting the response to ICBs of some specific tumors. So, we could easily apply the standard IHC staining antibody to test the MTHFR in tumors for research and clinic purpose. The results that antibody shows no positive IHC in specific cancer could help researchers to avoid wasting time and fees.

The limitations of our research is solely base on in-silico investigation, Experiments based on this study will be conducted in the future.

## Conclusion

Taken together, analysis of genomic, transcriptomic and clinic features comprehensively demonstrates statistical correlations between MTHFR and prognostic significance, immune infiltration, heterogeneity and stemness indices across tumor types, and beneficially understands the functions of MTHFR as a potential biomarker in immune infiltration, TME, tumorigenesis and ICBs from the perspective of clinical tumor samples. The finding could aid researchers to widen horizon in finding potential biomarker in cancer therapy and developing nascent drugs for better patients’ life.

## Data Availability

Data and download URLs involved in this study had been described in detail in the Methods section. All results generated in this study can be obtained by contacting the corresponding authors on reasonable request.

## Code availability

All the datasets analyses involved in this study were based on R software, and the used R packages were described in detail in the Methods section. The codes can be downloaded. from https://cran.r-project.org/ and https://www.rstudio.com/.
